# Bradykinin B_2_ Receptors of Dendritic Cells, Acting as Sensors of Kinins Proteolytically Released by Trypanosoma cruzi, Are Critical for the Development of Protective Type-1 Responses 

**DOI:** 10.1371/journal.ppat.0030185

**Published:** 2007-11-30

**Authors:** Ana Carolina Monteiro, Verônica Schmitz, Alexandre Morrot, Luciana Barros de Arruda, Fnu Nagajyothi, Alessandra Granato, João B Pesquero, Werner Müller-Esterl, Herbert B Tanowitz, Julio Scharfstein

**Affiliations:** 1 Instituto de Biofisica Carlos Chagas Filho, UFRJ, Rio de Janeiro, Brazil; 2 Intracellular Parasite Biology Section Laboratory of Parasitic Diseases, National Institute of Allergy and Infectious Diseases, Bethesda, Maryland, United States of America; 3 Instituto de Microbiologia Paulo de Goes, UFRJ, Rio de Janeiro, Brazil; 4 Albert Einstein College of Medicine, Bronx, New York, United States of America; 5 Departmento de Biofisica, USP, São Paulo, Brazil; 6 Institute of Biochemistry II, University of Frankfurt Medical School, Frankfurt, Germany; London School of Hygiene and Tropical Medicine, United Kingdom

## Abstract

Although the concept that dendritic cells (DCs) recognize pathogens through the engagement of Toll-like receptors is widely accepted, we recently suggested that immature DCs might sense kinin-releasing strains of Trypanosoma cruzi through the triggering of G-protein-coupled bradykinin B_2_ receptors (B_2_R). Here we report that C57BL/6.B_2_R^−/−^ mice infected intraperitoneally with T. cruzi display higher parasitemia and mortality rates as compared to B_2_R^+/+^ mice. qRT-PCR revealed a 5-fold increase in T. cruzi DNA (14 d post-infection [p.i.]) in B_2_R^−/−^ heart, while spleen parasitism was negligible in both mice strains. Analysis of recall responses (14 d p.i.) showed high and comparable frequencies of IFN-γ-producing CD4^+^ and CD8^+^ T cells in the spleen of B_2_R^−/−^ and wild-type mice. However, production of IFN-γ by effector T cells isolated from B_2_R^−/−^ heart was significantly reduced as compared with wild-type mice. As the infection continued, wild-type mice presented IFN-γ-producing (CD4^+^CD44^+^ and CD8^+^CD44^+^) T cells both in the spleen and heart while B_2_R^−/−^ mice showed negligible frequencies of such activated T cells. Furthermore, the collapse of type-1 immune responses in B_2_R^−/−^ mice was linked to upregulated secretion of IL-17 and TNF-α by antigen-responsive CD4^+^ T cells. In vitro analysis of tissue culture trypomastigote interaction with splenic CD11c^+^ DCs indicated that DC maturation (IL-12, CD40, and CD86) is controlled by the kinin/B_2_R pathway. Further, systemic injection of trypomastigotes induced IL-12 production by CD11c^+^ DCs isolated from B_2_R^+/+^ spleen, but not by DCs from B_2_R^−/−^ mice. Notably, adoptive transfer of B_2_R^+/+^ CD11c^+^ DCs (intravenously) into B_2_R^−/−^ mice rendered them resistant to acute challenge, rescued development of type-1 immunity, and repressed T_H_17 responses. Collectively, our results demonstrate that activation of B_2_R, a DC sensor of endogenous maturation signals, is critically required for development of acquired resistance to T. cruzi infection.

## Author Summary

Antibodies and IFN-γ-producing effector T cells are essential for the immune control of infection by Trypanosoma cruzi, the intracellular protozoa that causes human Chagas disease. Despite the potency of anti-parasite immunity, the parasites are not cleared from their intracellular niches. Instead, a low grade chronic infection prevails, provoking severe immunopathology in the myocardium. Although it is well established that innate sentinel cells sense T. cruzi through receptors for microbial structures, such as Toll-like receptors, it remained unclear whether endogenous inflammatory signals also contribute to the development of adaptive immunity. The present study was motivated by awareness that T. cruzi trypomastigotes (extracellular infective forms) are equipped with proteases that liberate the pro-inflammatory bradykinin peptide from an internal segment of kininogens. Here we demonstrate that splenic dendritic cells (DCs), the antigen-presenting cells that coordinate the adaptive branch of immunity in lymphoid tissues, are potently activated via G-protein-coupled bradykinin B_2_ receptors (B_2_R). Analysis of the outcome of infection in B_2_R-knockout mice revealed that the mutant mice developed a typical susceptible phenotype, owing to impaired development of IFN-γ-producing effector T cells. Notably, the immune dysfunction of B_2_R-knockout mice was corrected upon cell transfer of wild-type DCs, thus linking development of protective T cells to DCs' sensing of endogenous danger signals (kinins) released by trypomastigotes.

## Introduction

Chagas disease, the chronic cardiomyopathy caused by infection with the intracellular parasitic protozoan Trypanosoma cruzi, remains a major health problem in Central and South America [1]. Although acute Chagas disease may have a fatal outcome, the blood parasitemia, tissue parasite burden (liver, spleen, and heart), and the inflammatory sequel tend to subside with the onset of adaptive immunity. After several years of asymptomatic infection, approximately 30% of infected patients develop a chronic and progressive form of cardiomyopathy [2]. While not excluding a secondary pathogenic role for autoimmunity, studies in humans and animal models support the concept that parasite persistence in myocardial tissues is the primary cause of chronic immunopathology [3–6]. Cohort studies with chagasic patients have linked chronic heart pathology to T_H_1-type responses [7], but this proposition was recently called into question by a report indicating that the frequency of IFN-γ-producing effector/memory T cells is inversely correlated with the severity of chronic Chagas disease [8]. Animal model studies established that acquired resistance depends on development of serum antibodies as well as on IFN-γ-producing CD4^+^ and CD8^+^ T cells [9–12]. Recent studies indicated that CCR5 has a suceptible phenotype, attributed to impaired recruitment of effector T cells to parasitized heart tissues [13,14]. Although the dominant epitope specificities recognized by cytotoxic CD8 T cells are encoded by highly polymorphic genes [15], it is still unclear how T. cruzi escapes from immune surveillance [16–18].

In the present work, we set out to investigate the mechanims linking innate to adaptive immunity in the mouse model of T. cruzi infection. Early studies about innate resistance mechanisms indicated that macrophages upregulate nitric oxide (NO)-dependent trypanocydal responses [19] due to ligand-induced signaling of Toll-like 2 receptors (TLR2) [20,21] or TLR4 [22]. More recently, Bafica et al. reported that macrophages sense T. cruzi DNA via triggering of intracellular TLR9 [23]. Interestingly, they showed that acute infection is more severe in TLR2^−/−^ TLR9^−/−^ mice than in TLR9^−/−^ mice or either TLR2^−/−^- [23] or TLR4-deficient mice [22], albeit not as much as in the overtly susceptible MyD88^−/−^ mice [24]. While not formally excluding an additive innate role for TLR4, these collective studies suggested that cooperative activation of TLR2 and TR9 may account for the bulk of protective IFN-γ responses generated by MyD88-dependent signaling pathways [23,24]. Of note, analysis of macrophage activation by MyD88-independent pathways revealed that TLR/TRIF coupling promotes NO-dependent microbicidal responses through upregulation of type I interferons [25,26]. In spite of evidence that mice deficient in IL-12 [27] are highly susceptible to T. cruzi infection, it is still uncertain if induction of T_H_1-responses is strictly dependent on dendritic cell (DC) maturation by TLRs/MyD88-dependent pathways. Pertinently, it was reported that spleen cells from MyD88^−/−^ mice display small yet significant production of IL-12 and IFN-γ [24,28]. These observations imply that IL-12-dependent Th1 responses may be also controlled by MyD88-independent mechanisms, such as the NKT/CD1d pathway [29], or by endogenously released bradykinin (BK), an endogenous danger signal driving DC maturation [30–32].

“Kinins”, a small group of mediators related to the nonapeptide BK, activate immature DCs [30] as well as several other cell types through the binding to distinct subtypes of G-protein-coupled receptors: B_2_R (constitutive) and B_1_R (inducible) [33–36]. The B_2_R agonists, BK or lysyl-BK (LBK), are proteolytically excised from an internal segment of their parental (glyco)proteins, high or low molecular weight kininogens, by plasma or tissue kallikreins, respectively [33]. In the settings of infections, however, kinins can be generated through the direct action of microbial cysteine proteases, such as gingipain of Porphyromonas gingivalis [37] and cruzipain (CZP), the major cysteine protease of T. cruzi [38–41]. Using a subcutaneous model of T. cruzi infection, we recently demonstrated that trypomastigotes release kinins in peripheral tissues through the activity of CZP [31]. Once liberated from plasma borne–kininogens, the short-lived kinin peptides activate CD11c^+^DCs via B_2_R, inducing IL-12 production and stimulating the migration of these antigen-presenting cells (APCs) from the periphery to the draining lymph nodes, where they initiate T_H_1-like responses against T. cruzi [31,32]. Here we report that B_2_R-deficient mice infected intraperitoneally by T. cruzi display a typical susceptible phenotype. Adoptive cell transfer experiments demonstrate that CD11c^+^ DCs activated by the endogenous kinin/B_2_R-signaling pathway are critically required for the induction and/or maintenance of activated effector CD4^+^ and CD8^+^ T cells, while limiting the development of potentially detrimental IL-17-producing CD4^+^ T cell (T_H_17) responses in mice acutely infected with *T. cruzi.*

## Results

### Infection by the Intraperitoneal Route Discloses a Susceptible Phenotype in B_2_R**^−/−^** Mice

**Figure 1:**
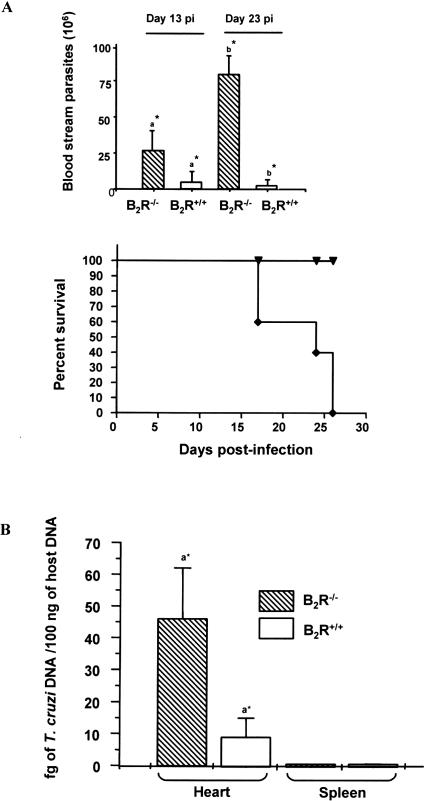
B_2_R^−/−^ Mice Are Susceptible to T. cruzi Infection by the Intraperitoneal Route (A) Temporal course of infection with the Dm28c T. cruzi strain in B_2_R^+/+^ and B_2_R^−/−^ mice. Parasitemia and survival curves of mice groups intraperitoneally infected with 1 × 10^6^ TCT of the Dm28c strain of T. cruzi. Parasitemia was evaluated with 5 μl of each infected mouse's blood in an optical microscope. Mortality was recorded daily. The data are representative of two independent experiments (*n* = 5 mice/group). Statistics were done by ANOVA and pair-wise comparisons were done by the Tukey test (a*, *p* < 0.05; b* *p* < 0.01). (B) Quantification of T. cruzi Dm28c in heart and spleen from infected animals as described above. qPCR was performed as described in Materials and Methods in 100 ng of total DNA at 14 d p.i. Bars represent an average of four to five animals per group ± SD. *p* < 0.05 between tissue parasitism in heart from wild-type and B_2_R^−/−^-infected animals as determined by Student *t* Test.

In order to test the hypothesis that kinins may contribute to immune control of T. cruzi infection [30,31], we injected intraperitoneally B_2_R^+/+^ C57BL/6 and B_2_R^−/−^ mice with tissue culture trypomastigotes (TCT) of either Dm28c strain (1 × 10^6^) or Brazil strain (1 × 10^4^). The data shown in Figure 1 indicate that wild-type mice infected with Dm28c TCT developed a low blood parasitemia and all the animals survived (Figure 1A, higher panel). In contrast, B_2_R^−/−^ mice infected with Dm28c showed a precocious blood parasitemia (day 13 post-infection [p.i.]), which further increased (approximately 3-fold) as the infection continued (23 d p.i.). Mortality rates indicated that B_2_R^−/−^ mice infected by Dm28c TCT started to die earlier (day 16) than wild-type mice and were all dead by day 27 (Figure 1A, lower panel). We then studied the outcome of infection with the Brazil strain. The results (Figure S1) show that wild-type mice displayed a relatively low blood parasitemia and the mortality rate did not exceed 20%. In contrast, the B_2_R^−/−^ mice infected by Brazil strain developed increased blood parasitemia, and 80% of these animals were dead by day 28 (Figure S1).

We then further characterized the outcome of intraperitoneal infection with the Dm28c strain, using a lower inoculum (6 × 10^5^). Analysis by real-time PCR (qPCR) showed that heart tissues of infected B_2_R^−/−^ mice (14 d p.i.) contained approximately 5-fold higher content of parasite DNA as compared to wild-type heart (Figure 1B). Surprisingly, we found that the parasite tissue burden in the spleen was very low both in B_2_R^+/+^ (0.30 ± 0.09 fg/100 ng host DNA) and B_2_R^−/−^ (0.46 ± 0.21 fg/100 ng host DNA) mice (Figure 1B). Thus, unlike the scenario observed in extra-lymphoid tissues, parasite outgrowth in the spleen is controlled by mechanisms that do not critically depend on activation of the kinin/B_2_R pathway, at least so at relatively early stages (14 d) of infection.

### Analysis of the Temporal Course of Type-1 Immune Responses in the Spleen

**Figure 2:**
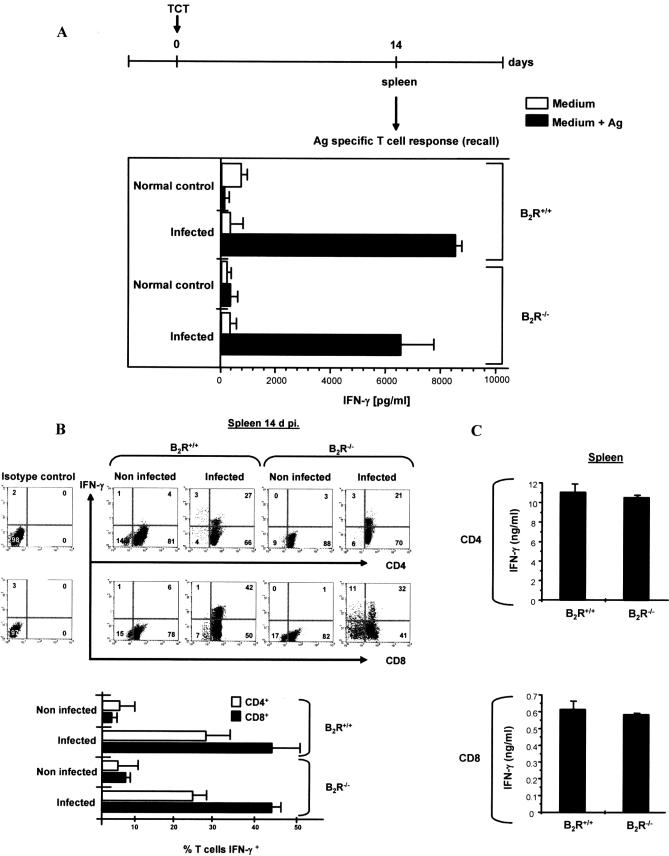
Splenic CD4^+^ and CD8^+^ T Cells from Infected B_2_R^+/+^ or B_2_R^−/−^ Mice Produce High Levels of IFN-γ at the Early Stage (14 d p.i.) of Infection Infections were performed by inoculation (intraperitoneally) of 6 × 10^5^ TCT (Dm28c). Spleens were removed from infected and non-infected B_2_R^+/+^ and B_2_R^−/−^ mice at 14 d p.i. (A) Assessment of IFN-γ production by splenocytes stimulated in vitro with T. cruzi Ag (25 μg/ml). (B) Purified CD4^+^ and CD8^+^ T cells were co-cultured with CD11c^+^ DCs loaded with T. cruzi Ag (25 μg/ml) for 18 h at 37 °C and were stained for CD4 or CD8 and IFN-γ as described in Materials and Methods. Dot plot profiles (*n* = 5 mice/group) are representative of results observed in four independent experiments. Column graphs (lower panel) indicate the mean ± SD of the frequency of IFN-γ-producing CD4^+^ or CD8^+^ T cells (*n* = 3). (C) Purified CD4^+^ and CD8^+^ T cells were co-cultured with CD11c^+^ DCs loaded with T. cruzi Ag (25 μg/ml) for 72 h at 37 °C, and supernatants were harvested and assayed for IFN-γ levels by ELISA. Values are the mean ± SD from one representative experiment with individual cells from five mice/group. Statistics were done by ANOVA and pair-wise comparisons were done by the Tukey tests.

Since the tissue parasitism in the spleen of wild-type and B_2_R^−/−^ mice (14 d p.i.) was marginal, we checked whether type-1 effector cells were generated in lymphoid tissues of both mice strains. Recall assays indicated that splenocytes from wild-type or B_2_R^−/−^ vigorously secreted IFN-γ upon stimulation with soluble T. cruzi antigen (Ag) (Figure 2A). Controls showed that, in the absence of T. cruzi soluble Ag, there was no significant production of IFN-γ by the splenocytes (Figure 2A). We then scrutinized the *ex vivo* recall responses of CD4^+^ or CD8^+^ T cells derived from either wild-type or B_2_R^−/−^ spleen (isolated from infected or naïve mice, as controls) using wild-type CD11c^+^ DCs (purified from normal spleen) as APCs, to exclude the possibility that eventual defects in Ag processing/presentation by B_2_R^−/−^ DCs could interfere with our “read-outs”. In keeping with the potent type-1 response elicited by unfractionated wild-type and B_2_R^−/−^ splenocytes (14 d p.i.), fluorescent activated cell sorting (FACS) analysis showed presence of high and comparable frequencies (Figure 2B, lower panel) of IFN-γ-producing CD4^+^ and CD8^+^ T cells in the spleens of wild-type and B_2_R^−/−^ mice (Figure 2B). Controls performed with Ag-stimulated CD4^+^ or CD8^+^ T cells isolated from naïve mice did not generate significant frequencies of IFN-γ-producing cells. Consistent with the similar FACS profiles, ELISA assays showed that IFN-γ was vigorously secreted by Ag-responsive splenic CD4^+^ or CD8^+^ T cells, irrespective of the mouse strain origin (Figure 2C).

**Figure 3:**
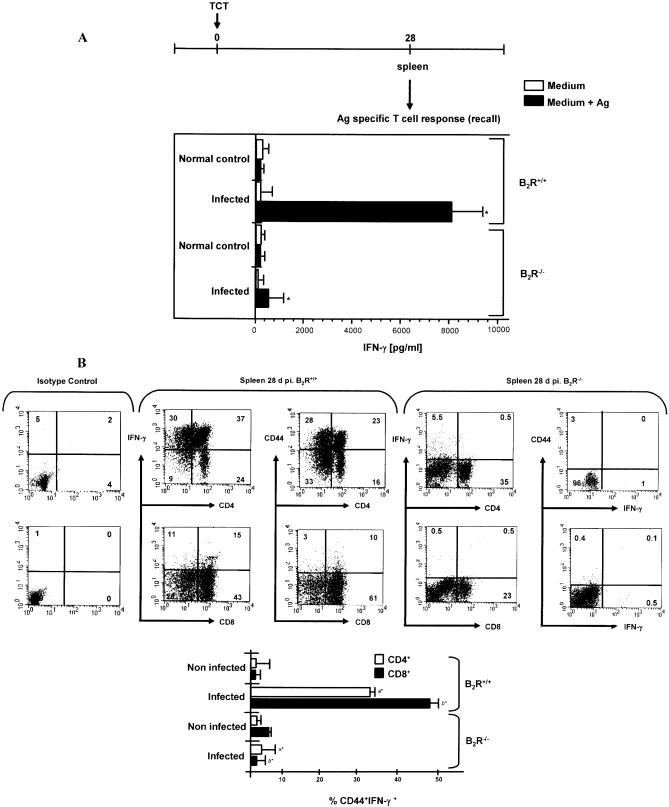
CD4^+^ and CD8^+^ T Cells from T. cruzi–Infected B_2_R^+/+^ Mice (28 d p.i.) Produce High Levels of IFN-γ (A) Assessment of IFN-γ production by splenocytes isolated from B_2_R^+/+^ and B_2_R^−/−^ mice infected at 28 d with Dm28c TCT. Cells were stimulated with T. cruzi Ag (25 μg/ml) for 72 h at 37 °C. Culture supernatants were harvested and assayed for IFN-γ. (B) T cells were stimulated with T. cruzi Ag (25 μg/ml) for 18 h at 37 °C and were stained for CD4 or CD8, IFN-γ, and CD44 marker as described in Materials and Methods. Cells from B_2_R^+/+^ mice were gated on CD4^+^IFN-γ^+^ or CD8^+^IFN-γ^+^ lymphocytes and examined for expression of CD44. Dot plot profiles (*n* = 5 mice/group) are representative of results observed in three independent experiments. Column graphs (lower panel) indicate the mean ± SD of the frequency of IFN-γ-producing CD4^+^CD44^+^ or CD8^+^CD44^+^ T cells (*n* = 3). Statistics were done by ANOVA and pair-wise comparisons were done by the Tukey test (*, *p* < 0.01).

We then checked if the presence of type-1 CD4^+^ and CD8^+^ effector T cells was maintained in the spleen as the infection continued. Recall assays performed 2 wk later (28 d p.i.) indicated that IFN-γ production by wild-type splenocytes remained vigorous, while the type-1 response of Ag-stimulated B_2_R^−/−^ splenocytes declined sharply (Figure 3A). We then repeated this analysis using CD4^+^ or CD8^+^ T cells purified from the spleens of infected wild-type mice or B_2_R^−/−^ mice, using wild-type DCs as APCs. Consistent with the data obtained with splenocytes, we found that Ag-stimulated T lymphocytes (CD4^+^ or CD8^+^) isolated from B_2_R^−/−^ spleen (28 d p.i.) secreted significantly lower levels of IFN-γ as compared to wild-type splenic T cells (unpublished data). We then performed FACS analysis to further characterize the phenotypic changes that occurred in the spleen, as the acute infection advanced (28 d p.i.). Our results (Figure 3B) showed that Ag-stimulated T cells isolated from wild-type spleen showed high frequencies of IFN-γ-producing CD4^+^ and CD8^+^ T lymphocytes. Moreover, a significant fraction of activated CD4^+^ and CD8^+^ T cells isolated from spleen of wild-type infected mice displayed the CD44 surface marker. As expected, addition of Ag to CD4^+^ or CD8^+^ T cell cultures from naïve mice did not lead to IFN-γ production (Figure 3B, lower panel). In contrast, B_2_R^−/−^ spleen presented low frequencies of IFN-γ-producing CD4^+^ or CD8^+^ effectors (CD44^−^) (Figure 3B). Although we have no direct evidence that the Ag-responsive T cells detected *ex vivo* include functionally active effectors, it is worthwhile mentioning that adoptive transfer of CD4^+^/CD8^+^ T cells (isolated from wild-type mice at 60 d p.i.) into B_2_R^−/−^ mice rendered these recipient mice resistant to lethal infection (0% mortality, *n* = 5; three independent experiments), as compared to non-manipulated B_2_R^−/−^ mice (100% mortality) or B_2_R^−/−^ mice that received CD4^+^/CD8^+^ T cells from normal wild-type mice (100% mortality).

### Type-1 Responses by Intracardiac CD4^+^ and CD8^+^ T Cells from B_2_R^−/−^ mice Are Compromised at Early Stages of Infection

**Figure 4:**
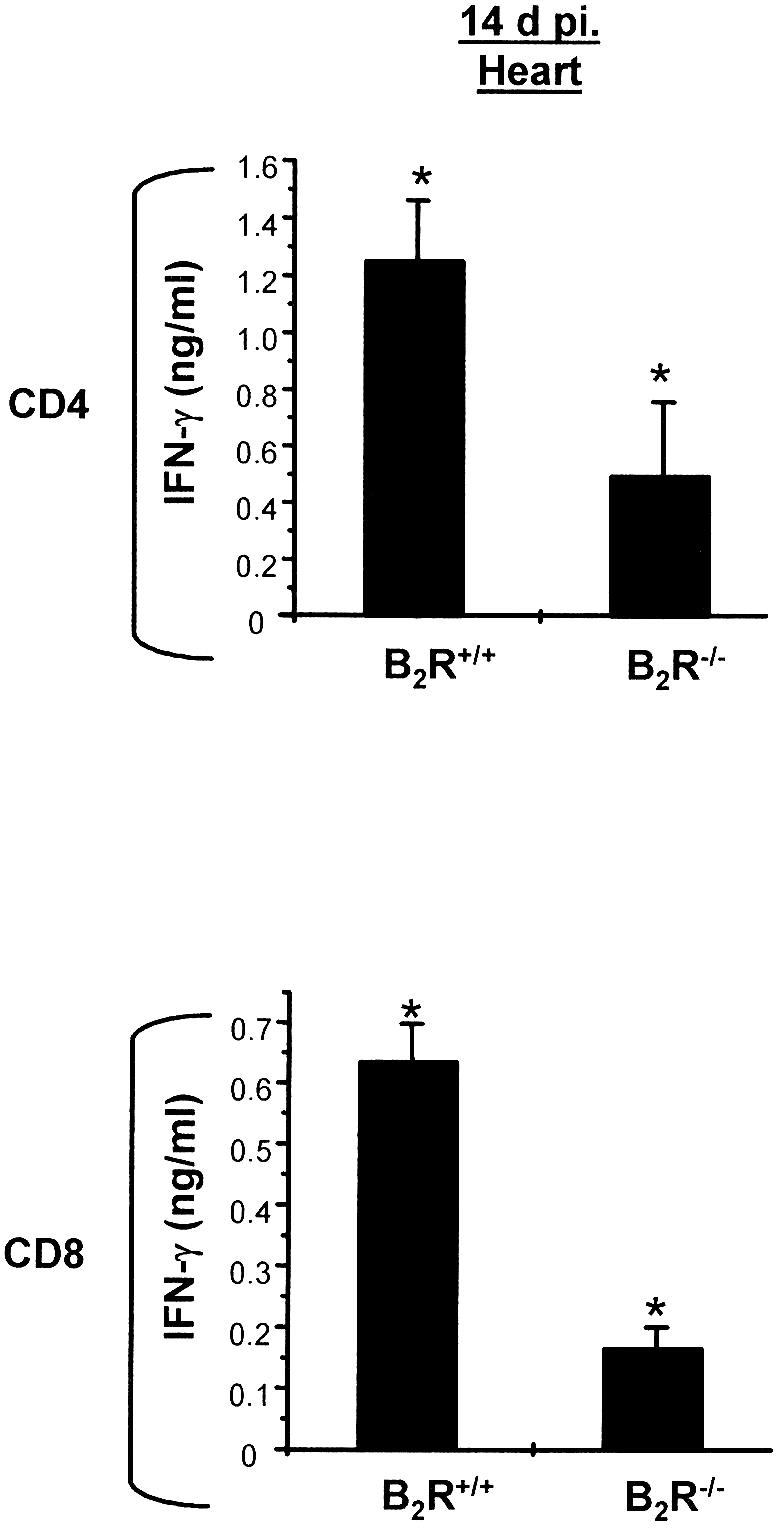
B_2_R^−/−^-Infected Mice Present Lower Frequencies of IFN-γ-Producing Intracardiac CD4^+^ and CD8^+^ T Cells at the Early Stage (14 d p.i.) of Infection Assessment of IFN-γ production by heart-derived CD4^+^ and CD8^+^ T cells isolated (14 d p.i.) from B_2_R^+/+^ and B_2_R^−/−^ mice. Cells were co-cultured with CD11c^+^ DCs loaded with T. cruzi Ag (25 μg/ml) for 72 h at 37 °C. Culture supernatants were harvested and assayed for IFN-γ by ELISA. Data are representative of two independent experiments (*n* = 5 mice/group). Statistics were done by ANOVA and pair-wise comparisons were done by the Tukey test (*, *p* < 0.05) .

As mentioned earlier in this section, we found a 5-fold increase of T. cruzi DNA in the heart of B_2_R-deficient mice at day 14 p.i., as compared to wild-type heart (Figure 1C). In view of these findings, we set out to determine if cardiac tissues of wild-type and B_2_R^−/−^ mice contained type-1 effector T cells. Recall assays (again using wild-type splenic CD11c^+^ DCs as APCs) showed that IFN-γ production by intracardiac B_2_R^−/−^ CD4^+^ T cells was significantly diminished (over 50%) as compared to responses elicited by experienced CD4^+^ T lymphocytes isolated from wild-type heart at 14 d p.i. (*p* < 0.01) (Figure 4). Similarly, the initial recall response of intracardiac CD8^+^ T cells isolated from B_2_R^−/−^ mice was approximately 60% lower than that of wild-type CD8^+^ T cells (Figure 4).

**Figure 5:**
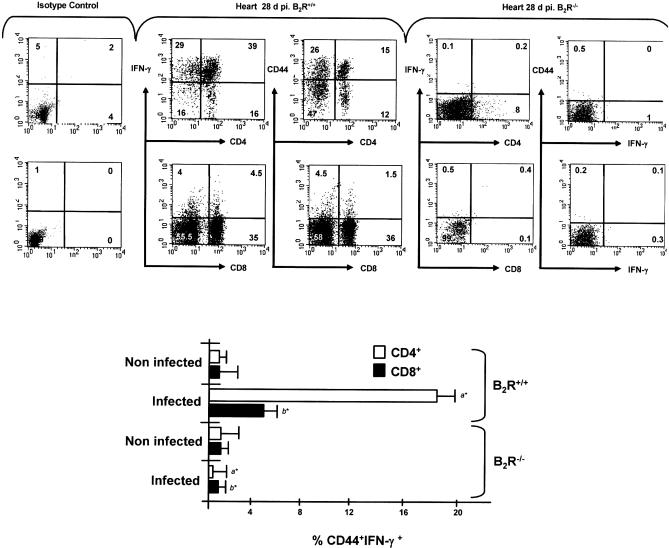
B_2_R^−/−^-Infected Mice Display Negligible Frequencies of Heart-Derived CD4^+^ and CD8^+^-Activated T Cells at Advanced Stages of Acute Infection Heart-derived T cells were stimulated with T. cruzi Ag (25 μg/ml) for 18 h at 37 °C and were stained for CD4 or CD8, IFN-γ, and CD44 marker as described in Materials and Methods. Cells from B_2_R^+/+^ mice were gated on CD4^+^IFN-γ^+^ or CD8^+^IFN-γ^+^ lymphocytes and examined for expression of CD44. Dot plot profiles (*n* = 5 mice/group) are representative of results observed in two independent experiments. Column graphs (lower panel) indicate the mean ± SD of the frequency of IFN-γ-producing CD4^+^CD44^+^ or CD8^+^CD44^+^ T cells (*n* = 2). Statistics were done by ANOVA and pair-wise comparisons were done by the Tukey tests (*, *p* < 0.01).

We then checked if the type-1 cytokine response of intracardiac T cells from B_2_R^−/−^ mice was further compromised as the infection continued. The FACS profiles of wild-type-infected mice (28 d p.i.) revealed high frequencies of IFN-γ-producing intracardiac CD4^+^ and CD8^+^ T cells (Figure 5). In addition, we found that the CD44 marker characteristic of activated T cells was present in a significant proportion of wild-type intracardiac CD4^+^ T cells, and (to lower extent) also in the CD8^+^ T cell subset (Figure 5, upper and lower panels). In contrast, B_2_R^−/−^ mice exhibited very low frequencies of CD4^+^ and CD8^+^ T cells in the intracardiac CD3^+^ T cell pool at day 28 p.i. (Figure 5). Following the same trend, IFN-γ-producing CD4^+^ or CD8^+^ effector T cells, and activated phenotypes (CD44^+^CD4^+^ and CD44^+^CD8^+^ T cells) were virtually absent from B_2_R^−/−^ heart. Collectively, these results suggest that activation of the endogenous kinin/B_2_R signaling pathway in T. cruzi–infected mice may have an impact on the control mechanisms affecting the temporal and spatial activity of type-1 effectors.

### The Depressed T_H_1 Response of B_2_R^−/−^ Infected Mice Is Inversely Correlated with Increased Production of IL-17 and TNF-α

**Figure 6:**
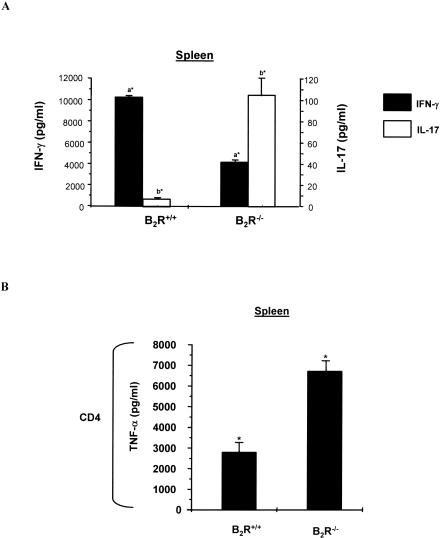
The Depressed T_H_1 Response of B_2_R^−/−^-Infected Mice Is Inversely Correlated with Increased Production of IL-17 Splenic CD4^+^ T cells, isolated from infected B_2_R^+/+^ and B_2_R^−/−^ mice (28 d p.i.). T cells were co-cultured with CD11c^+^ DCs pulsed with T. cruzi Ag (25 μg/ml) for 72 h at 37 °C. Culture supernatants were harvested and assayed for IFN-γ and IL-17 (A) and TNF-α (B) levels by ELISA. Values are the mean ± SD from one representative experiment with individual cells from five mice/group. Statistics were done by ANOVA and pair-wise comparisons were done by the Tukey test (a*, *p* < 0.05; b*, *p* < 0.01; *, *p* < 0.05).

Considering that the type-1 responses of B_2_R^−/−^ mice were depressed both in the heart (as early as 14 d p.i.) and spleen (28 d p.i.), we then asked if these effects were coupled to T_H_2 upregulation. Our results indicated that Ag-stimulated T CD4^+^ T cells (isolated from B_2_R^−/−^ heart or spleen) did not upregulate IL-4 production (unpublished data). Since IFN-γ inhibits T_H_17 lineage development in vitro [42,43], we wondered if the reduced T_H_1 responses observed in B_2_R^−/−^ mice were accompanied by rises of IL-17- and TNF-α-producing T cells. Recall responses made at 28 d p.i. (Figure 6A) revealed that splenic CD4^+^ T lymphocytes from wild-type mice did not secrete significant levels of IL-17, while splenic B_2_R^−/−^ CD4^+^ T cells upregulated IL-17. The same trend was found when we measured TNF-α levels secreted by experienced B_2_R^−/−^ CD4^+^ T cells (Figure 6B). Similar data were obtained when we compared Ag-stimulated responses of intracardiac CD4^+^ T cells isolated from B_2_R^−/−^ versus wild-type mice, as discussed later in this section. Collectively, these data suggest that the T_H_17/T_H_1 ratio was drastically increased as the acute infection advanced in the highly susceptible B_2_R^−/−^ mice.

### TCT Induce IL-12 Production by Splenic CD11c^+^ DCs via B_2_R

**Figure 7:**
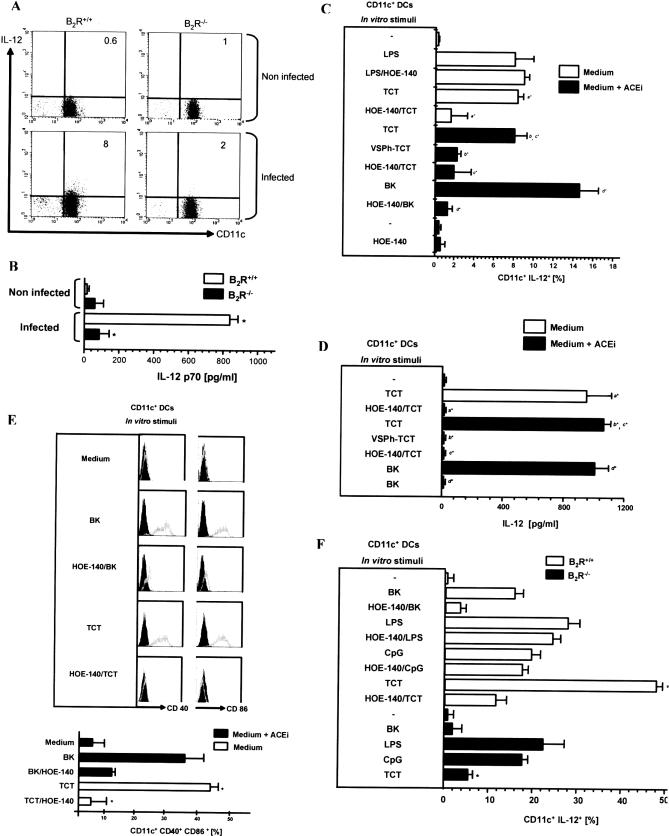
CD11c^+^ DCs Sense TCT via the Kinin/B_2_R Activation Pathway (A) IL-12 production by splenic CD11c^+^ DCs of infected mice. B_2_R^+/+^ and B_2_R^−/−^ male mice were infected with 1 × 10^6^ TCT intravenously. Non-infected animals served as control. CD11c^+^ DCs were isolated from spleen of infected mice at 18 h p.i. and cultured in RPMI complete medium. FACS profiles were done with CD11c-FITC and anti-IL12-PE (*n* = 6 mice/group). (B) ELISA determination of IL-12 production by CD11c^+^ DCs from B_2_R^+/+^ and B_2_R^−/−^ mice non-infected and infected with 1 × 10^6^ TCT intravenously. Statistics were done by ANOVA and pair-wise comparisons were done by the Tukey test (*, *p* < 0.01). (C) Intracellular IL-12 produced by splenic CD11c^+^ DCs of BALB/c mice incubated in vitro with 10^6^ TCT or VSPh-TCT, ratio DC/TCT (1:3), in the presence or absence of 25 μM lisinopril (ACEi) for 18 h, and brefeldin A was added in the final 4 h. In same experiments, 10 nM BK was added to the medium, with or without 0.1 μM HOE-140, 10 μM VSPh, or 10 ng/ml LPS, as indicated. FACS profiles were done with CD11c-FITC and anti-IL12-PE. Each bar represents the % of DCs producing IL-12. Data represent the mean ± SD from six independent experiments. Statistics were done by ANOVA and pair-wise comparisons (represented by ^*a*, *b*, *c*, *d*^) were done by the Tukey test (*, *p* < 0.05). (D) IL-12 levels in supernatants from splenic CD11c^+^ DC cultures, as above. Statistics were done by ANOVA and pair-wise comparisons (represented by ^*a*, *b*, *c*, *d*^) were done by the Tukey test (*, *p* <0.01). (E) Histograms for CD40 and CD86 expression in splenic CD11c^+^ DCs. Gray lines represent labeling by anti-CD40-FITC or anti-CD86-FITC, and bold black lines represent labeling by isotype-matched control mAb (rat IgG_2a_-FITC). Dot plot profiles (*n* = 5 mice/group) are representative of results observed in at least three independent experiments. Column graphs (lower panel) indicate the mean ± SD of the frequency of CD11c^+^ CD40^+^ CD86^+^ DCs. Statistics were done by ANOVA and pair-wise comparisons were done by the Tukey test (*, *p* < 0.05). (F) ELISA determination of IL-12 production by splenic CD11c^+^ DCs from B_2_R^+/+^ versus B_2_R^−/−^ mice stimulated with TCT. Controls were done with BK (10 nM) and HOE-140 (0.1 μM) in the presence of 25 μM of ACEi (lisinopril). Controls were also performed with LPS (10 ng/ml) and CpG (100 ng/ml) in the presence or absence of HOE-140 (0.1 μM). Data represent the mean ± SD from three independent experiments done in triplicate each. Statistics were done by ANOVA and pair-wise comparisons were done by the Tukey tests (*, *p* < 0.05).

Since type-1 responses were impaired in infected B_2_R^−/−^ mice, we sought to determine if IL-12 responses were preserved, or not, in these mutant mice. To this end, we inoculated Dm28c TCT (1 × 10^6^) intravenously in wild-type and B_2_R^−/−^ mice, isolated splenic CD11c^+^ DCs 18 h p.i., and measured IL-12 production by FACS. The results (Figure 7A) showed a marked increase in the frequency of IL-12-producing CD11c^+^ DCs (8%) in B_2_R^+/+^ in relation to non-infected controls (no IL-12 staining). In contrast, splenic CD11c^+^ DCs isolated from infected B_2_R^−/−^ mice showed a low frequency (2%) of IL-12-positive cells (Figure 7A). These results were corroborated by ELISA determinations of IL-12 responses produced by DCs isolated from intravenously infected mice (Figure 7B). Of note, we found that macrophages (CD11b^+^ F4/80^+^) from infected wild-type and B_2_R^−/−^ mice show enhanced production of IL-12 as compared to naïve mice, suggesting that alternative mechanisms (i.e., B_2_R-independent) may govern IL-12 production by splenic macrophages (unpublished data). Extending these in vivo studies to BALB/c mice, these animals were pre-treated, or not, with the B_2_R antagonist HOE-140 before intravenous injection of TCT. The FACS profiles showed a sharp increase of IL-12-positive CD11c^+^ DCs in BALB/c mice injected with either TCT (Figure S2) or BK (positive control) (Figure S2). In contrast, BALB/c mice pre-treated with HOE-140 showed a reduced frequency of IL-12-positive CD11c^+^ DCs (Figure S2). Collectively, the data indicate that B_2_R drives IL-12 production by splenic DCs, at least at very early stages of the infection.

We then carried out in vitro studies to verify if the parasites could induce the maturation of CD11c^+^ (splenic) DCs through the activation of the kinin/B_2_R signaling pathway. IL-12 production and surface expression of co-stimulatory proteins were used as read-out for DC maturation. FACS analyses showed that CD11c^+^ DCs (BALB/c) did not produce significant IL-12 levels in the absence of parasites (Figure 7C). In contrast, IL-12 production was drastically increased upon addition of exogenous BK (positive control) or TCT, whereas HOE-140 cancelled both stimuli (Figure 7C). Notably, TCT induced IL-12-producing DCs irrespective of the presence/absence of lisinopril, a rather selective inhibitor angiotensin-converting enzyme (ACEi) (Figure 7). Specificity controls confirmed that HOE-140 did not interfere at all with the magnitude of IL-12 responses induced by lipopolysaccharide (LPS) (Figure 7C). In agreement with the FACS data, ELISA determinations of IL-12 levels in cultures supplemented with HOE-140 confirmed that TCT activate immature DCs through B_2_R (Figure 7D). Controls in the absence of pathogen indicated that lisinopril or HOE-140 as such did not induce IL-12 production by DCs (Figure 7C). Additionally, DCs cultivated with either TCT or BK (positive control) displayed increased surface expression of CD40 and CD86 (Figure 7E). Of note, HOE-140 cancelled the phenotypic changes induced by TCT (Figure 7E, upper and lower panels), while responses induced by BK were significantly reduced by this B_2_R antagonist (Figure 7E, lower panel).

Since TCT generate kinins via CZP while invading endothelial cells, we next asked if parasite cysteine proteases were required for DC activation. This question was addressed by pre-incubating TCT with methylpiperazine-Phe-homoPhe-vinylsulfone-benzene (VSPh), an irreversible inhibitor of CZP. After washing the VSPh-TCT, they were added to DC cultures. Whether using FACS and ELISA, we found that VSPh-TCT failed to drive significant IL-12 production by DCs (Figure 7C and 7D), adding weight to the concept that the parasite relies on CZP to generate the innate kinin stimuli.

In order to verify whether the B_2_R^−/−^ CD11c^+^ DCs were fully capable of responding to TLR agonists, we compared the in vitro response profile induced by cytosine-phosphate-guanine (CpG) and LPS. As shown in Figure 7F, IL-12 responses were of the same magnitude as compared to wild-type C57BL/6 DCs. Moreover, HOE-140 did not interfere with wild-type DC responsiveness to CpG and LPS (Figure 7F). Notably, the magnitude of B_2_R^−/−^ DC response to TCT was nearly 10% of IL-12 responses observed in wild-type CD11c^+^ DCs (Figure 7F). As expected, TCT or BK elicited vigorous IL-12 production in CD11c^+^ DCs from wild-type mice. In both cases, the IL-12 response was partially blocked by HOE-140 (Figure 7F). In contrast, BK did not induce IL-12 in B_2_R^−/−^ DCs (Figure 7F).

### Adoptive Transfer of CD11c^+^ DCs from B_2_R^+/+^ into Susceptible B_2_R^−/−^ Mice Restored Host Capability to Control Infection through Induction of Type-1 Effector T Cells

**Figure 8:**
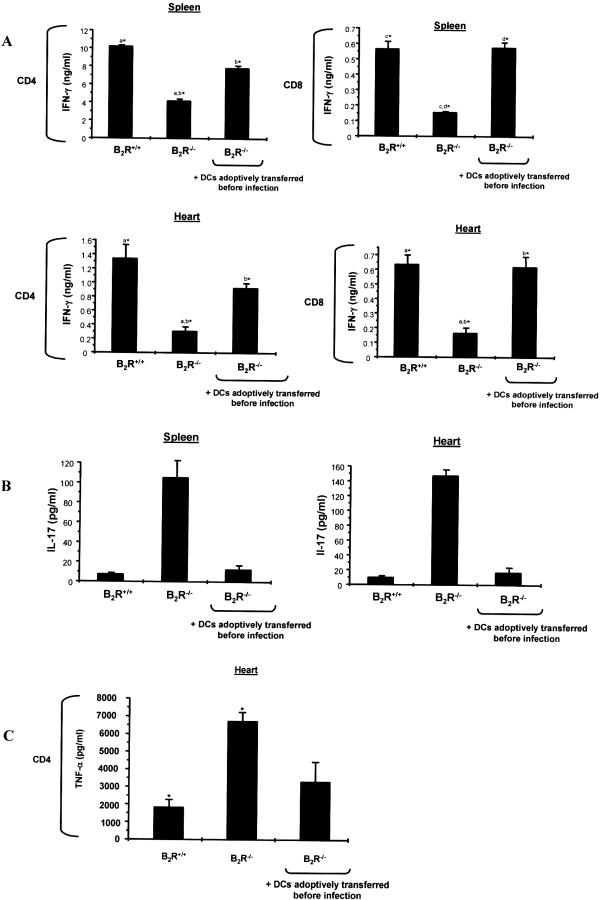
Adoptive Transfer of B_2_R^+/+^ CD11c^+^ DCs into Susceptible B_2_R^−/−^ Mice Restores Type-1 Immunity Assessment of cytokine production by splenic and heart-derived CD4^+^ and CD8^+^ T cells isolated (28 d p.i.) from B_2_R^+/+^, B_2_R^−/−^, and B_2_R^−/−^ recipient mice. The transfer of CD11c^+^ DCs (spleen B_2_R^+/+^) into B_2_R^−/−^ mice was carried out by the intravenous route. Purified T cells were co-cultured with CD11c^+^ DCs loaded with T. cruzi Ag (25 μg/ml) for 72 h at 37 °C. Culture supernatants were harvested and assayed for IFN-γ (A), IL-17 (B), and TNF-α (C) levels by ELISA. Values are the mean ± SD from one representative experiment with individual cells from five mice/group. Statistics were done by ANOVA and pair-wise comparisons were done by the Tukey test (*, *p* < 0.05).

As mentioned earlier, we found that production of IFN-γ by Ag-experienced CD4^+^ and CD8^+^ T cells from B_2_R^−/−^ spleen and heart declined sharply as the infection continued (28 d p.i.). In view of those findings, we asked whether the deficient type-1 responses of B_2_R^−/−^ mice were restored upon adoptive transfer of wild-type DCs. To address this question, we adoptively transferred (intravenously) immature B_2_R^+/+^ CD11c^+^ DCs (10^6^ cells) into B_2_R^−/−^ mice before injection of the parasites. As controls, recipient B_2_R^−/−^ mice received an equivalent number of CD11c^+^ DCs isolated from donor B_2_R^−/−^ spleen. As expected, our controls showed that B_2_R^−/−^ mice succumbed (100% mortality, *n* = 5; three independent experiments) at day 30. In contrast, 100% of the B_2_R^−/−^ recipient mice reconstituted with B_2_R^+/+^ DCs survived the acute challenge. Of note, the mice of the specificity control group (B_2_R^−/−^ DCs → B_2_R^−/−^ mice) succumbed (100%) to the infection, thus ruling out the possibility that adaptive immune function was restored due to non-specific activation of these APCs during the DC isolation procedure. We then ran another set of experiments to verify if the DC transfer maneuver had restored (type-1) acquired immunity of B_2_R^−/−^ recipient mice. Recall assays performed at day 28 p.i. confirmed that splenic or intracardiac (CD4^+^ or CD8^+^) T cells from control B_2_R^−/−^ mice secreted lower levels of IFN-γ as compared to experienced CD4^+^ or CD8^+^ T cells isolated from B_2_R^+/+^ spleen or heart (Figure 8A). Notably, B_2_R^−/−^ mice that received adoptive transfer of B_2_R^+/+^ DCs recovered the ability to generate IFN-γ-producing CD4^+^ and CD8^+^ T cells (Figure 8A). Conversely, the DC transfer to B_2_R^−/−^ mice repressed the secretion of IL-17 (Figure 8B) and TNF-α (Figure 8C) by Ag-experienced (splenic or intracardiac) CD4^+^ T cells of the reconstituted B_2_R^−/−^ mice, therefore simulating the phenotype of wild-type-infected mice.

## Discussion

In the present work, we have demonstrated that the immune dysfunction of B_2_R^−/−^ mice infected intraperitoneally with T. cruzi is a consequence of defective sensing of endogenously released kinins by immature CD11c^+^ DCs. Our analysis of the adaptive immune responses of infected B_2_R^−/−^ appointed a role for the kinin signaling pathway in the development of type-1 effector T cells. The critical importance of DCs as sensors of kinins was confirmed by adoptive cell transfers (wild type DC→ B_2_R^−/−^ mice), which reversed the susceptible phenotype of B_2_R^−/−^ mice while restoring the development of type-1 effector T cells, both in the spleen and cardiac tissues of recipient B_2_R^−/−^ mice.

The notion that the kinin-releasing trypomastigotes induce DC maturation through B_2_R is supported by the following experimental evidence. First, our in vitro studies showed that TCT vigorously induced IL-12 responses in splenic DCs originating from wild-type (C57BL/6) mice, while failing to activate B_2_R^−/−^ DCs. Second, we demonstrated that HOE-140, a specific antagonist of B_2_R, efficiently blocked DC maturation (IL-12 induction, upregulation of CD80, CD86, and CD40). Furthermore, the irreversible inhibitor of CZP (K11777) mitigated the IL-12 stimulatory activity (B_2_R-driven) of TCT, thus implicating the major cysteine protease of T. cruzi in the kinin generation mechanism. Extending these observations to the in vivo settings, we then analyzed IL-12 production by splenic CD11c^+^ DCs isolated 18 h after systemic inoculation (intravenously) of Dm28c TCT. Experiments performed with BALB/c mice showed that mice pre-treated with HOE-140 presented reduced frequencies of splenic CD11c^+^ IL-12^+^ DCs. Adding weight to these results, we demonstrated that TCT induced high frequencies of CD11c^+^ IL-12^+^ DCs in wild-type (C57BL/6) spleen, while failing to evoke significant IL-12 responses in DCs isolated from B_2_R^−/−^ spleen. Notably, preliminary studies indicated that macrophages (CD11b^+^F4/80^+^) isolated from the spleen of these wild-type and B_2_R^−/−^ mice develop comparable IL-12 responses. Given that type-1 immune responses in the spleen of B_2_R^−/−^ mice are well preserved at day 14 p.i., it is possible that macrophages activated by alternative routes provide the IL-12 signals that drive adaptive immunity in this secondary lymphoid tissue.

Although we cannot claim that conventional DCs are the primary or even unique in vivo targets of T. cruzi in the spleen, the above mentioned results support the concept that kinin-releasing pathogens may drive DC maturation in vivo through the activation of G-protein-coupled B_2_ receptors [32]. Since lymphoid tissues are irrigated by non-fenestrated capillaries, we may predict that trypomastigotes invading the splenic stroma are faced with an abundant supply of blood-borne proteins, such as kininogens. Given biochemical evidence that interactions of high molecular weight kininogens with heparan sulfate proteoglycans potentiate the kinin-releasing activity of CZP [40], it is plausible that the extracellular trypomastigotes might promptly liberate these paracrine signaling peptides while moving through extracellular matrices, hence driving DC maturation via B_2_R [31,32].

At first sight, our finding that TCT induce DC maturation via the endogenous kinin/B_2_R pathway appears to conflict with the well-established concept that innate sentinel cells sense pathogens via pattern recognition receptors (PRRs), such as the members of the TLR family [28,44]. Indeed, early studies of macrophage (IFN-γ-primed) interaction with T. cruzi (Y strain) suggested that TLR2 and TLR4 ligands [20–22] are major drivers of innate responses in T. cruzi infection. In a limited attempt to investigate the functional relationship of B_2_R and TLRs, we examined the outcome of TCT interaction in vitro with CD11c^+^ DCs (splenic origin) derived from either TLR2^−/−^ or TLR4^d/d^ mice. Our results indicated that TCT induced vigorous IL-12 responses both in TLR2^−/−^ DCs and TLR4^d/d^ DCs (unpublished data). Moreover, we found that addition of HOE-140 to the TCT/DC culture system blocked IL-12 responses by TLR2^−/−^ or TLR4^d/d^ DCs (unpublished data). Admittedly, complementary studies with DCs from double TLR2/TLR4 knockout mice and MyD88^−/−^ mice are required to rule out the possibility that B_2_R-responsive phenotypes of TLR2^−/−^ DCs and TLR4^d/d^ DCs reflect compensatory responses, respectively induced by TLR4 and TLR2 ligands of T. cruzi [20–22]. The intertwined nature of the innate pathways controlling IL-12 production by APCs is illustrated by the recent demonstration [23] that T. cruzi DNA potently induces IL-12 production by mouse macrophages through the activation of TLR9. Given the evidence that DCs are parasitized by T. cruzi [45], it will be interesting to determine if endogenous (BK/LBK) and exogenous (T. cruzi DNA) danger signals may activate their respective sensor receptors, B_2_R and TLR9, at distinct temporal stages (i.e., early and late) of intracellular infection.

While examining the frequencies of type-1 effectors in extra-lymphoid and lymphoid tissues of wild-type and B_2_R^−/−^-infected mice, we became aware that B_2_R deficiency affected the temporal and spatial distribution of IFN-γ-producing CD4^+^ and CD8^+^ T cells. Recall assays performed at day 14 p.i. revealed weakened IFN-γ production by intracardiac CD4^+^ and CD8^+^ T cells isolated from B_2_R^−/−^ mice. However, we found high and comparable frequencies of INF-γ-producing T cells in the spleen of the same B_2_R^−/−^ and wild-type mice. Since the parasites are scarcely found in the spleens of wild-type and B_2_R^−/−^ mice, we may infer that activation of the kinin/B_2_R pathway is dispensable for early induction of type-1 effectors in the spleen. Adoptive cell transfer studies are required to find out if the induction of these early type-1 effector T cells is controlled by MyD88-coupled pathways [24], such as those triggered by TLR2/TLR9 [23] and/or by IL-1R/IL-18 R [44]. In addition, it is possible that IL-12 induction by the NKT/CD1 pathway [29] may also contribute to early development of type-1 effectors in lymphoid tissues.

It is intriguing that intracardiac CD4^+^ and CD8^+^ T cells from B_2_R^−/−^ mice (14 d p.i.) showed impaired production of IFN-γ, despite the fact that the spleen of these mice displayed high frequencies of type-1 effectors. Coincidently, tissue parasite burden is drastically increased in B_2_R^−/−^ heart, thus showing an inverse correlation between these two parameters at day 14 p.i. Although we cannot a priori assume that Ag specificities of T cells recruited to the heart of wild-type and B_2_R^−/−^ mice at 14 d p.i. are necessarily the same, independent studies performed with the Brazil [46] and Y strain of T. cruzi [47] converged in appointing cytotoxic CD8^+^ T cells as the key effectors controlling intracellular parasite outgrowth in cardiac tissues. So far, efforts to characterize the Ag specificity of intracardiac CD8^+^ T cells in our infection model have been hampered by the findings that Dm28c T. cruzi strain did not present open reading frames for genes coding for ASP-2 antigens [48], which in other systems provide dominant epitopes recognized by cytotoxic CD8^+^ T cells [46,47]. In spite of these limitations, it is conceivable that immunoregulatory dysfunctions were responsible for the weakened type-1 responses observed in peripheral T cells from B_2_R^−/−^ mice. For example, it is possible that the migratory competence of effector T cells generated in lymphoid tissues may depend on DC activation via the kinin/B_2_R pathway. Pertinently, recent analysis of the susceptible phenotype of CCR5^−/−^ mice infected with T. cruzi implicated this chemokine receptor in the recruitment of CD8^+^ and CD4^+^ effector T cells into infected heart [13,14]. Given these precedent findings, it will be worthwhile investigating if B_2_R and CCR5 signaling, whether acting separately or in conjunction, might promote the migration of effector T cells to peripheral sites of infection, such as the heart.

As the infection advanced (14→28 d), wild-type mice developed high frequencies of IFN-γ-producing CD4^+^ and CD8^+^ effector T cells, both in the spleen and heart. Interestingly, a significant proportion of these Ag-responsive T cells displayed activated (CD44^+^) phenotypes. In contrast, B_2_R^−/−^ mice showed negligible frequencies of activated type-1 effectors at day 28, both in spleen and heart. Of note, we found that the intracardiac CD4^+^ and CD8^+^ T populations recovered from the CD3^+^ pool of B_2_R^−/−^ mice were significantly contracted (Figure 5). Considering that B_2_R^−/−^ mice recovered the capacity to mount protective type-1 responses upon adoptive transfer of wild-type DCs, it is possible that maintenance of T cell homeostasis may depend, at least to some degree, on DC responses elicited by endogenously released kinins. Albeit speculative, this hypothesis is worth exploring in light of independent reports showing that aberrant T cell apoptosis is the primary cause of the immunoregulatory abnormalities underlying host susceptibility to acute infection by the Dm28c strain of T. cruzi [49].

Another intriguing phenotypic characteristic of infected B_2_R^−/−^ mice emerged when we monitored production of IL-17 and TNF-α in our recall assays. Unexpectedly, we found that the weakened T_H_1 responses of B_2_R^−/−^ CD4^+^ T cells (whether isolated from the spleen/heart) at day 28 d p.i. was accompanied by upregulated production of IL-17 and TNF-α, two pro-inflammatory cytokines associated with the effector activity of T_H_17 cells. Recently characterized as a separate lineage of pro-inflammatory T helper cells distinct from conventional T_H_1 and T_H_2 cells [42,43], T_H_17 cells differentiate from naïve precursors under the critical influence of IL-6 and TGF-β1 [50]. It is also known that committed T_H_17 cells depend on the IL-23 survival signal to develop their pro-inflammatory function in vivo [51]. Notably, at early stages of infection (14 d p.i.), there was no significant production of IL-17 and TNF-α by spleen- or heart-derived T cells from infected B_2_R^−/−^ mice, whether detected by conventional recall assays or polyclonal activation with anti-CD3 antibodies (unpublished data). It is unclear why the T_H_1/T_H_17 balance was inverted as the acute infection progressed in B_2_R^−/−^ mice. Recently, IL-27 was identified as the cytokine that suppresses T_H_17 differentiation driven by IL-6 and TGF-β via STAT-1, independently of IFN-γ [50]. Interestingly, T. cruzi–infected WSX-1 mice (deficient in the IL-27Ra) [52] develop severe hepatic injury, correlating with overproduction of various pro-inflammatory cytokines, such as IL-6, TNF-α, and IFN-γ [52]. Although T_H_17 responses were not evaluated in T. cruzi–infected WSX-1 mice, these animals strongly upregulated T_H_2 cytokines [52]. However, we were unable to detect IL-4 production or IgG isotype switching in infected B_2_R^−/−^ mice, indicating that these mice strains do not share the same phenotype. Importantly, the recovery of type-1 responses in DC recipient B_2_R^−/−^ mice was associated with reduced production of IL-17 and TNF-α. Additional studies are underway to determine if DCs activated by the kinin/B_2_R pathway may influence T_H_1/T_H_17 lineage development in T. cruzi infection via IL-27, or through alternative mechanisms.

Collectively, our results have linked development of acquired resistance to T. cruzi infection to DC functional responses controlled by the kinin/B_2_R signaling pathway. Our study provides a paradigm for investigations of the innate role of endogenously released kinin “danger” signals in T_H_1/T_H_17 development in other infections and inflammatory diseases.

## Materials and Methods

### Mice and parasites.

Experiments were done with mouse strains BALB/c, C57BL/6 WT (B_2_R^+/+^), and C57BL/6 B_2_R^−/−^ [53]. TCT (Dm28c clone of T. cruzi) were harvested from the supernatants of infected LLC-MK2 cultures maintained in Dulbecco's Modified Eagle Medium (DMEM) supplemented with 2% FCS. Freshly released parasites were washed 3X with excess PBS before being used in experiments. Epimastigotes (EPI) of Dm28c clone of T. cruzi were cultivated in standard liver infusion tryptose medium (LIT) containing 10% FCS (GIBCO). Where indicated, TCT were pre-incubated for 20 min at RT with 10 μM of VSPh.

### In vitro activation of CD11c^+^ DC isolated from normal mouse spleen.

Splenic DCs were isolated with anti-CD11c magnetic beads (Miltenyi Biotec) and stimulated in vitro with TCT (3 × 10^6^/well) in DMEM/10% fresh FCS for 18 h at 37 °C in the presence or absence of 25 μM lisinopril (Lis; Sigma), an inhibitor of the angiotensin converting enzyme (ACEi) and/or 0.1 μM HOE-140, as indicated. In some experiments, DCs were treated with VSPh-TCT. Controls were done with 10 nM BK, 10 ng/ml LPS, or 100 ng/ml CpG. For intracellular staining of IL-12, 1 × 10^6^ DCs were washed and pre-incubated with 2% of normal mouse serum (NMS) supplemented with anti-mouse CD16/CD32 FCγ III/II receptor (clone 2.4G2) (1 μg/10^6^ cells) (BD Biosciences). The washed cells were stained with anti-mouse CD11c-FITC (BD Biosciences) in PBS/2% NMS. After washing (2X PBS), the cells were fixed in 2% paraformaldehyde, washed, and permeabilized with 0.05% saponin (Sigma-Aldrich). Staining with PE-labeled anti-IL-12 p40/p70 (BD Biosciences) was performed in PBS/2% NMS/0.5% saponin. Surface expression of co-stimulatory proteins was monitored by incubating DCs with antibodies to CD40 or CD86 (BD Biosciences) in the presence of PBS/2% NMS. Isotype-matched control was performed with rat IgG-FITC or IgG_1_-PE (BD Biosciences). Samples were analyzed by (FACSCalibur) (BD Biosciences), and data analyses were done with CELLQuest software (BD Biosciences) or Win-MDI software (TSRI).

### Isolation and characterization of DCs from mice infected with T. cruzi.

Mice were pre-treated or not with 10 mg/kg intraperitoneally of ACEi (captopril) and/or 100 μg/kg subcutaneously of HOE-140, as indicated, and 1 h later the mice were injected intravenously with 1 × 10^6^ TCT. DCs were isolated from spleen at 18 h p.i. Briefly, pooled lymph node fragments were treated with collagenase D (Sigma-Aldrich), and CD11c^+^ DCs were positively selected using magnetic beads covered with anti-mouse CD11c (Miltenyi Biotec; 90% pure). CD11c^+^ DCs (10^6^ cells/well) were incubated for 4 h in RPMI complete medium with 10 μg/ml brefeldin A (Sigma-Aldrich) and were stained for CD11c and IL-12 p40/p70 as described above.

### Cytokine production by spleen- and heart-derived T cells isolated from T. cruzi–infected mice.

B_2_R^+/+^ and B_2_R^−/−^ mice were infected by the intraperitoneal route with 6 × 10^5^ TCT. After 28 d, splenocytes were recovered and were stimulated with 25 μg/ml boiled soluble T. cruzi antigen (EPI-Ag). Total CD3^+^ T cells (T cell Enrichment column; R&D Systems) were purified from either spleen or heart of infected B_2_R^+/+^ or B_2_R^−/−^ mice (14 d and 28 d p.i.). CD4^+^ and CD8^+^ T cells were also purified (14 d and 28 d p.i.) from spleen or heart of infected mice with magnetic microbeads conjugated to anti-mouse CD4^+^ and CD8^+^ (Miltenyi Biotec) and isolated by passing over a MACs LS^+^ column held in a VarioMACS magnetic separator (Miltenyi Biotec). Positively selected cells were 85%–95% pure, as determined by flow cytometry analysis. Recall assays were performed by co-culturing 1 × 10^6^ CD3^+^, CD4^+^, or CD8^+^ T cells with 1 × 10^4^ splenic CD11c^+^ DCs from B_2_R^+/+^ mice as APCs loaded with 25 μg/ml boiled soluble T. cruzi antigen (EPI-Ag). Culture supernatants were collected after 72 h and cytokines (IFN-γ, IL-17, TNF-α) were quantified by ELISA utilizing purified and biotinylated Abs (R&D Systems). Values are presented as pg cytokine/ml (mean ± SD). Statistical differences between mean values were evaluated by ANOVA, and pair-wise comparisons were done by the Tukey test.

### Flow cytometry.

B_2_R^+/+^- and B_2_R^−/−^-infected mice were killed at the time points indicated (14 d and 28 d p.i.) and single cell suspensions were prepared from the spleen and heart. Red blood cell–depleted cells were stimulated with 25 μg/ml boiled soluble T. cruzi antigen (EPI-Ag) and treated with anti-mouse CD16/CD32 FCγ III/II receptor before staining. Cells were then fixed in 2% paraformaldehyde and stained with FITC-labeled mouse antibody against CD4 or CD8, and PE-Cy-labeled mouse antibody against CD44 (BD Biosciences). For intracellular staining, stimulated cells were treated with brefeldin A (BD Biosciences) and stained with PE-labeled anti-IFN (XMG1.2; eBiosciences). Samples were analyzed by FACSCalibur (BD Biosciences), and data analyses were done with CELLQuest software (BD Biosciences).

### Quantification of tissue parasite loads by qPCR.

qPCR for parasite quantification was performed as described previously [54] with minor modifications. Briefly, DNA was isolated from spleen and heart tissues of B_2_R^+/+^ and B_2_R^−/−^ mice infected by the intraperitoneal route with 6 × 10^5^ TCT, after digestion with proteinase K, followed by a phenol-chloroform-isoamyl alcohol affinity extraction. q-PCR using 100 ng of total DNA was performed on an ABI PRISM 7900 sequence detection system (Applied Biosystems) using SYBR Green PCR Master Mix according to the manufacturer's recommendations. Purified T. cruzi DNA (American Type Culture Collection) was sequentially diluted for curve generation in aqueous solution containing equivalent amounts of DNA from uninfected mouse tissues. The equivalence of host DNA between samples was normalized by levels of genomic *beta-2 microglobulin* (*B2m*) gene in the same samples. The following primers were used for T. cruzi genomic DNA, TCZ, GCTCTTGCCCACACGGGTGC (forward), and CCAAGCAGCGGATAGTTCAGG (reverse); and for genomic *B2m*, CTGAGCTCTGTTTTCGTCTG (forward) and TATCAGTCTCAGTGGGGGTG (reverse).

### Quantitative determination of IFNγ levels by qPCR.

B_2_R^+/+^ and B_2_R^−/−^ mice were infected with 1 × 10^4^ trypomastigotes of the Brazil strain. Hearts were obtained at 15 d and 30 d p.i. RNA from the tissues was isolated using the Trizol LS reagent following the manufacturer's protocol. Briefly, 5 ng of RNA was reverse-transcribed in a final volume of 20 μl using Superscipt II transcriptase (Invitrogen). The reverse transcription mixture consisted of 1 mM dNTPs (Pharmacia Biotech), 20 mM dithiothreitol, 50 mM Tris HCl (pH 8.3), 75 mM KCl, 3 mM MgCl2, 2 ng hexamer (Pharmacia Biotech), and 200 U of superscript RT RNase H- reverse transcriptase (Invitrogen). The reaction was incubated for 50 min at 42 °C. The qPCR primers for IFN-γ were 5′ forward GCGGCCTAGCTCTGAGACAA and 5′ reverse GACTGTGCCGTGGCAGTAAC, which amplified the 97-bp IFN-γ gene fragment. qPCR was carried out using magnesium chloride (2 mM), primers, and the PCR Sybr Green Master Mix (Roche Applied Science) in a final volume of 20 μl. The reaction conditions for qPCR used for quantification of IFN-γ have been previously described [55]. A standard curve for the quantification of IFN-γ was developed in the range of 0.5 pg pg using the primers at same conditions. The result was normalized using GAPDH mRNA for each sample. The primer sequence and the conditions used for the real-time PCR quantification were the same as previously published [55].

## Supporting Information

Figure S1 B_2_R^−/−^ Mice Are Also Susceptible to Brazil T. cruzi Strain Infection by the Intraperitoneal Route Temporal course of infection with the Brazil T. cruzi strain in B_2_R^+/+^ and B_2_R^−/−^ mice. Parasitemia and survival curves of mice groups intraperitoneally infected with 1 × 10^4^ TCT of the Brazil strain of T. cruzi. Parasitemia were evaluated with 5 μl of each infected mouse's blood in an optical microscope. Mortality was recorded daily. The data are representative of two independent experiments (*n* = 10 mice/group).
(949 KB TIF)

Figure S2 Immature DCs Sense TCT via the Kinin/B_2_R Activation Pathway **(**A) IL-12 production by splenic CD11c^+^ DCs of infected mice. BALB/c male mice were pre-treated or not with HOE-140 (100 μg/kg) for 1 h prior to injection of 1 × 10^6^ TCT intravenously. Non-infected ACEi-treated animals served as control. CD11c^+^ DCs were isolated from spleen of infected BALB/c at 18 h p.i. and cultured in RPMI complete medium. FACS profiles were done with CD11c-FITC and anti-IL12-PE. Each bar represents the % of DCs producing IL-12 beyond threshold levels. Data represent the mean ± SD from two independent experiments (*n* = 6 mice/group). Statistics were done by ANOVA and pair-wise comparisons (represented by ^*a*, *b*, *c*, *d*^) were done by the Tukey test (*, *p* < 0.05).
(870 KB TIF)

## Abbreviations

ACEi: angiotensin-converting enzyme inhibitor
Ag: antigen
APC: antigen-presenting cell
BK: bradykinin
BR: bradykinin receptor
CpG: cytosine-phosphate-guanine
CZP: cruzipain
DC: dendritic cell
EPI: epimastigote
FACS: fluorescent activated cell sorting
LPS: lipopolysaccharide
p.i.: post-infection
PRR: pattern recognition receptor
qPCR: real-time PCR
TCT: tissue culture trypomastigotes
T_H_17: IL-17-producing CD4^+^ T cells
TLR: Toll-like receptor
VSPh: methylpiperazine-Phe-homoPhe-vinylsulfone-benzene
