# Supplementary figures and images for: Bradykinin B2 Receptors of Dendritic Cells, Acting as Sensors of Kinins Proteolytically Released by Trypanosoma cruzi, Are Critical for the Development of Protective Type-1 Responses
Source: PLoS Pathog. 2007 Nov 30;3(11):e185. doi: 10.1371/journal.ppat.0030185 (PMC2098834; doi:10.1371/journal.ppat.0030185)

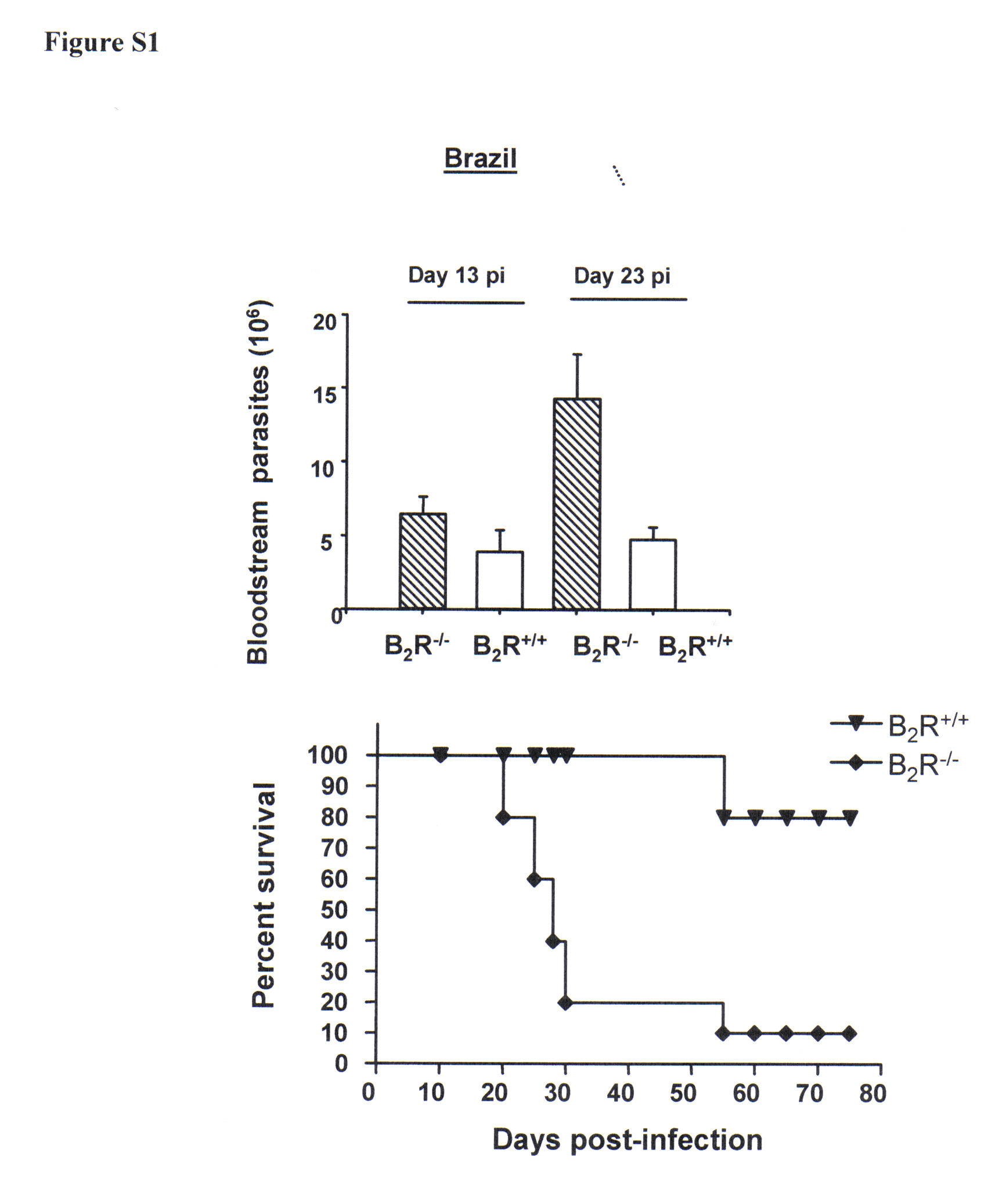

Supplement: Figure S1 — Temporal course of infection with the Brazil T. cruzi strain in B2R+/+ and B2R−/− mice. Parasitemia and survival curves of mice groups intraperitoneally infected with 1 × 10^4^ TCT of the Brazil strain of T. cruzi. Parasitemia were evaluated with 5 μl of each infected mouse's blood in an optical microscope. Mortality was recorded daily. The data are representative of two independent experiments (n = 10 mice/group). (949 KB TIF) [file ppat.0030185.sg001.tif]

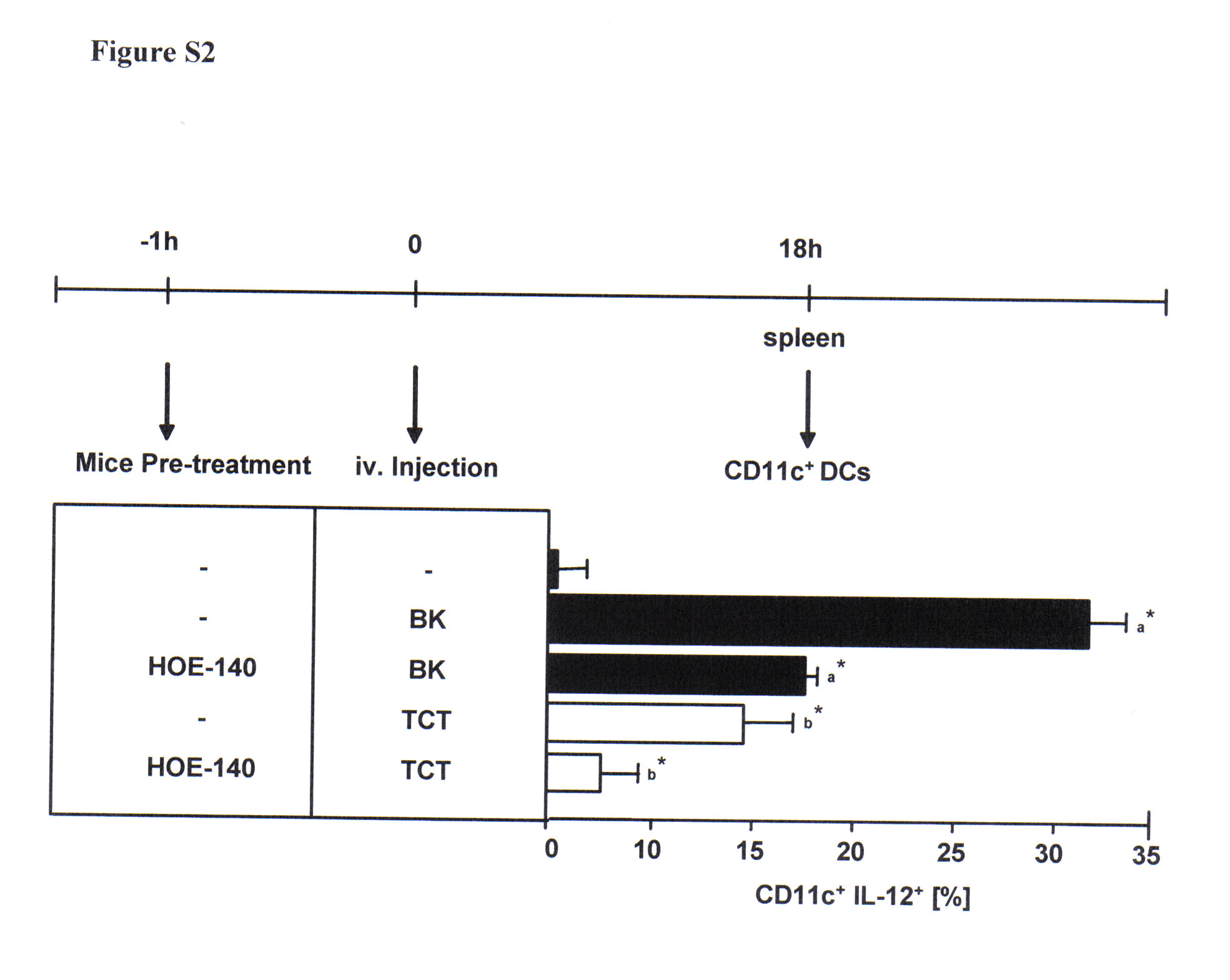

Supplement: Figure S2 — (A) IL-12 production by splenic CD11c+ DCs of infected mice. BALB/c male mice were pre-treated or not with HOE-140 (100 μg/kg) for 1 h prior to injection of 1 × 10^6^ TCT intravenously. Non-infected ACEi-treated animals served as control. CD11c+ DCs were isolated from spleen of infected BALB/c at 18 h p.i. and cultured in RPMI complete medium. FACS profiles were done with CD11c-FITC and anti-IL12-PE. Each bar represents the % of DCs producing IL-12 beyond threshold levels. Data represent the mean ± SD from two independent experiments (n = 6 mice/group). Statistics were done by ANOVA and pair-wise comparisons (represented by a, b, c, d) were done by the Tukey test (*, p < 0.05). (870 KB TIF) [file ppat.0030185.sg002.tif]
